# Deep brain stimulation for dystonia in Finland during 2007–2016

**DOI:** 10.1186/s12883-019-1370-y

**Published:** 2019-06-24

**Authors:** Rebekka M. Ortiz, Filip Scheperjans, Eero Pekkonen

**Affiliations:** 1 Department of Neurology, Helsinki University Hospital, Haartmaninkatu 4, 00029 HUS Helsinki, Finland; 2 Department of Clinical Neurosciences (Neurology), University of Helsinki, Helsinki, Finland

**Keywords:** Deep brain stimulation, Dystonia, Generalized dystonia, Focal dystonia

## Abstract

**Background:**

Dystonia is a movement disorder substantially affecting the quality of life and the ability to work. A proportion of patients does not respond to first line pharmacotherapy. Deep brain stimulation (DBS) is established as a primary operative treatment option for severe drug resistant dystonia. We studied dystonia patients treated with DBS in Finland between the years 2007–2016 to evaluate the use and outcomes of DBS treatment.

**Methods:**

We analysed the hospital records of dystonia patients, who underwent DBS operation during 2007–2016 in Finland. The clinical and technical parameters were recorded as well as preoperative assessments and treatments. The response to DBS was evaluated retrospectively using the Global Dystonia Rating Scale (GDS).

**Results:**

Out of 585 dB implantations during the study period, 37 were done for dystonia. The clinical response improved significantly with time in the isolated focal dystonia group, and at 12 months, 22 of 32 patients had over 50% alleviation of the GDS score. There was only one subclinical intracerebral haemorrhage, and four infections leading to revision. Speech impairment and limb coordination problems were common stimulation- related adverse events and were mostly resolved or relieved with the adjustment of stimulation parameters.

**Conclusions:**

DBS seems to be beneficial in dystonia. Although DBS is indicated for dystonia in Finland, the number of operations did not increase at the same rate as DBS operations in general. DBS appears to be a safe and effective treatment for focal as well as generalized dystonia.

**Electronic supplementary material:**

The online version of this article (10.1186/s12883-019-1370-y) contains supplementary material, which is available to authorized users.

## Background

Dystonia is a heterogeneous group of disorders, characterized by sustained or intermittent muscle contraction causing abnormal postures and/or repetitive movements with unknown aetiology [1]. It is the third most common movement disorder after essential tremor and Parkinson’s disease (PD) considerably affecting the quality of life and ability to work [2].

Currently there are only symptomatic treatments for dystonia, the most effective being oral treatment with anticholinergic drugs for generalized dystonia and intramuscular botulinum neurotoxin (BoNT) treatment for focal dystonia, specifically for cervical dystonia, as the first line treatment [3]. However, several patients do not respond to BoNT treatment or they lose the effect over time. Further, the response to oral pharmacotherapy, used particularly in generalized dystonia, is frequently inadequate and has several side effects [4].

High-frequency deep brain stimulation (DBS) targeting bilaterally globus pallidus interna (GPi) has been established as effective second line treatment in medically refractory dystonia with severely impaired quality of life [5–9]. DBS is considered a safe alternative to pallidotomy because of its reversibility, lower complication risk for bilateral procedures and adjustment possibilities [10]. DBS is recommended for generalized and segmental primary dystonia (level A recommendation) and in medical-refractory focal dystonia (level B) [10, 11]. In acquired dystonia, DBS is considered less effective, with the exception of tardive dystonia (level C) [11].

The aim of this study was to evaluate the use and outcomes of dystonia DBS in Finland. We specially focused on indications, response and adverse events. Using patient databases and medical records, we retrospectively studied dystonia patients treated with DBS in Finland between the years 2007–2016.

## Methods

We retrospectively analysed the hospital records of patients, who were diagnosed with dystonia (ICD-10 diagnoses G24.1-G24.9) and underwent DBS operation between 1.1.2007 and 31.12.2016 at four out of five DBS centres in Finland. The data of fifth centre with four patients was not available. Data of four patients were removed from further analysis. One patient had essential tremor, one had Tourette syndrome and two patients were excluded because DBS was removed 1 week after operation for unknown reasons.

The following data was obtained from the files: patient age on operation day, maximum follow up time, gender, the day of DBS operation, re-operations and removal of DBS, the type of the DBS generator, stimulation parameters at six and 12 months, the centre where operated, diagnosis and classification of dystonia, the duration of symptoms before operation, whether patient had neuropsychological or psychiatric evaluation, if the patient was able to work after operation, previous BoNT injections, and whether the BoNT injections continued after DBS. The device and stimulation –related perioperative and long-term adverse events during maximum follow-up time were analysed. Adverse events were categorized into adverse events needing no action or resolving with programming and serious adverse events requiring hospital admission. No exact target coordinates were available from patient records.

The motor response to DBS was retrospectively estimated from patient records at six and 12 months after primary DBS operation using the Global Dystonia Severity Rating Scale (GDS) [12]. The GDS score was evaluated from different body areas from 0 to 10 (0 being no dystonia in body area, 1 minimal dystonia, 5 moderate dystonia, 10 most severe dystonia). The 10 areas tested were: eyes and upper face; lower face; jaw and tongue; larynx; neck; shoulder and proximal arm; distal arm and hand including elbow; pelvis and upper leg; distal leg and foot; trunk.

Due to non-normal distribution of the data, Mann-Whitney U -test was used to compare continuous independent factors including generator parameter values and GDS score between groups and Wilcoxon signed ranks tests for related factors including GDS score in different time points. Spearman correlation coefficient was used for correlation analyses between generator parameters and generator life. Bonferroni correction was used to account for multiple comparisons. *p* < 0.05 was considered statistically significant. Normality testing was done using the Shapiro-Wilk test. The statistical analysis was done using SPSS version 24.0 (SPSS Inc., Chicago, IL, USA).

## Results

Thirty-seven patients received DBS because of dystonia during the years 2007–2016 in Finland with a gradual annual increase of operations (Additional file 3: Figure S1). Twenty patients were operated in Helsinki, eight in Tampere, six in Oulu and three in Kuopio university hospitals. There was a total of 77 operations including primary implantations, generator changes and electrode revisions and removals. During the years 2007–2016, altogether 585 dB operations were performed, and dystonia DBS accounted on average for 6,4% of all DBS operations. The clinical characteristics of patients are summarized in Table 1.

**Table 1:** The clinical characteristics of DBS patients

	Focal	Generalized	Segmental	Hemidystonia	All
	(*n* = 21)	(*n* = 10)	(*n* = 5)	(*n* = 1)	(*n* = 37)
Female	13 (62%)	6 (60%)	3 (60%)	1 (100%)	23 (62%)
Male	8 (38%)	4 (40%)	2 (40%)		14 (38%)
Mean age at operation ± SD, years	51,9 ± 6,6	45,7 ± 19,3	58,9 ± 8,5	25,7	50,3 ± 12,9
Mean follow-up time ± SD, months	19 ± 13	19 ± 16	25 ± 18	96	22 ± 19
Etiology					
Idiopathic	21 (100%)	8 (80%)	5 (100%)		34 (92%)
Inherited		1 (10%)			1 (3%)
Acquired		1 (10%)		1 (100%)	2 (5%)
Nervous system pathology					
No evidence	21 (100%)	5 (50%)	4 (80%)		30 (81%)
Structural lesion		3 (27%)		1 (100%)	4 (11%)
Degeneration		2 (18%)	1 (20%)		3 (8%)
Clinical characteristics					
Associated features					
Isolated dystonia	21 (100%)	5 (50%)	4 (80%)		30 (81%)
combined dystonia				1 (100%)	1 (3%)
Other neurological manifestation		5 (50%)	1 (20%)		6 (16%)
Disease course					
Stabile	20 (95%)	3 (30%)	2 (40%)	1 (100%)	26 (70%)
Progressive	1 (5%)	7 (70%)	3 (60%)		11 (30%)
Age at onset					
3–12 years		5 (50%)			5 (14%)
13–20 years	2 (9%)	1 (10%)			3 (8%)
21–40 years	10 (48%)	1 (10%)		1 (100%)	12 (32%)
over 40 years	9 (43%)	3 (30%)	5 (100%)		17 (46%)
Symptoms before DBS					
under 10 years	9 (43%)	5 (50%)	4 (80%)	1 (100%)	19 (51%)
over 10 years	12 (57%)	5 (50%)	1 (20%)		18 (49%)
Working ability before DBS					
Fit to work, full-time	7 (33%)	3 (30%)	1 (20%)		11 (30%)
Unable to work	11 (52%)	4 (40%)	2 (40%)	1 (100%)	19 (51%)
Retired because of age	3 (14%)	3 (30%)	2 (40%)		8 (19%)

All focal dystonia patients had isolated cervical dystonia. Of five segmental dystonia patients, one had cranio-cervical dystonia, three cervico-brachial dystonia, and one had severe blepharospasm with PISA-syndrome associated with Parkinson’s disease. One patient had acquired hemidystonia and chorea after stroke. Of generalized dystonia patients, one had DYT-1 gene mutation and the two had positive family history without known gene mutation. One had neurodegeneration with brain iron accumulation (NBIA), one had generalized dystonic tremor and one had acquired tardive dystonia.

Before DBS, eleven patients were considered fit to work. Nineteen patients were either on a long sick leave or retired because of sickness and eight patients were retired because of age. Only three patients who were on a long sick leave with cervical dystonia returned to work after DBS.

In most cases, neither neuropsychological nor psychiatric evaluation was done (25 patients). Preoperative neuropsychological evaluation was performed in nine patients. Nine patients received psychiatric evaluation and five patients received both evaluations. Eleven patients had previously been diagnosed with depression, and five of them had preoperative psychiatric evaluation. Postoperatively, one patient with known depression reported worsening of symptoms. Two patients, with no diagnosis of depression, reported postoperative significant depression. Two patients had preoperative memory problems, one with NBIA, but were evaluated in neuropsychological tests as eligible for DBS. In one patient cognitive problems worsened after DBS, probably due to the progression of NBIA. Both patients with previous cognitive problems had only minor benefit from DBS.

**Fig. 1:**
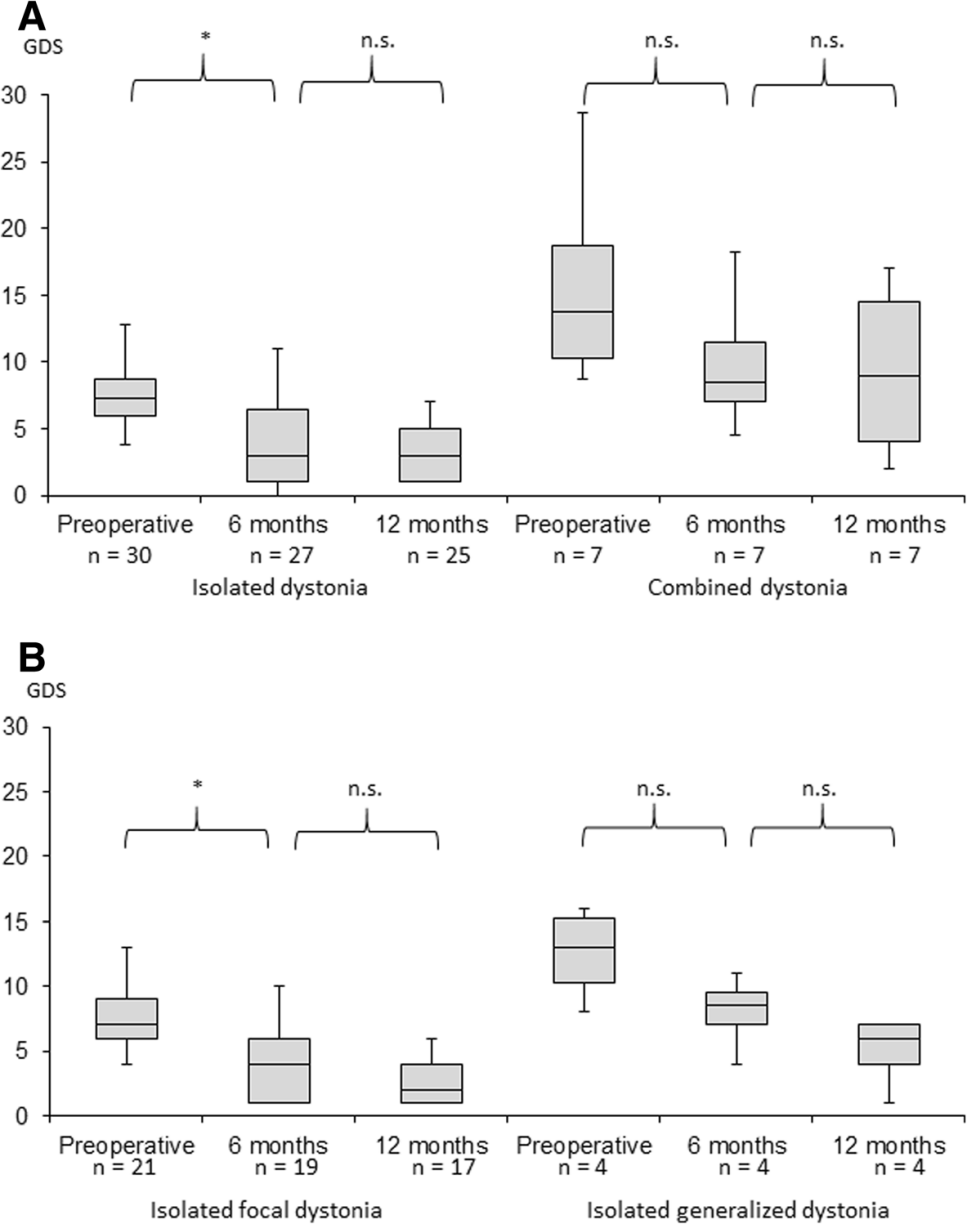
Box-and-whisker-plot of clinical response estimated by GDS. **a** The GDS preoperatively and at six and twelve months, respectively, in isolated and combined dystonia. **b** The GDS preoperatively and at and twelve months, respectively, in isolated focal and generalized dystonia. The box represents inter-quartile range, middle line represents median and upper and lower whiskers extend to 95th and 5th percentile. * = *p* < 0.005. n.s. = not significant

The clinical response as evaluated with GDS score improved significantly between preoperative state and at 6 months after operation in the isolated dystonia group (*p* < 0.005, Wilcoxon signed ranks test). The improvement of the GDS score continued at 12 months even though the difference between six and 12 months was not significant. At 12 months, the mean reduction in GDS score was 55% and 22 of 32 patients had over 50% alleviation. Out of 10 patients with less benefit, 6 had mediocre response with stimulation related side effects. Only 4 patients did not benefit at all, one of them had NBIA, one had other progressive generalized dystonia and two had marked side effects with increased stimulation. In the heterogeneous combined dystonia group, the results varied more and no significant improvement was seen (Fig. 1a). In the combined dystonia group, the patient with post-stroke hemidystonia had the best response with 75% drop in GDS score. The preoperative mean GDS was 7,3 ± 2,1 in isolated focal dystonia patients and 12,5 ± 3,7 in isolated general dystonia patients reflecting the different clinical phenotype of these groups. The clinical response improved significantly with isolated focal dystonia patients, the reduction in GDS score was 65% between preoperative score and 12 months. A similar non-significant trend was seen with isolated general dystonia patients (60% reduction in GDS score) (Fig. 1b).

The clinical response as measured with GDS at 6 and 12 months with isolated dystonia patients did not differ between age groups under vs. over 50 years, duration of disease under vs. over 10 years, or patients with vs. without previously diagnosed depression (Additional file 1: Table S1).

All patients had had preoperative brain MRI to calculate electrode trajectory and target. Microelectrode recording (MER) was used in all but the largest centre (18/38 patients) to pinpoint the target. Postoperatively, the correct electrode positioning was confirmed by brain computer tomography in all patients and evaluated by the operating neurosurgeon in each center.

All patients had primarily non-rechargeable dual-channel Medtronic Activa PC implantable pulse generator (IPG), except one patient, who had Medtronic Kinetra IPG. One patient had primarily temporary pulse generator that was replaced after 2 months with permanent IPG (Activa PC). Quadripolar electrodes were located bilaterally in GPi, except in one patient with acquired hemidystonia, who had unilateral combined VIM and GPi electrodes. Contacts were selected according to mark points based on stereotactic positioning on MRI/CT fusion scans and/or MER in the beginning and adjusted later according to clinical response. There was no significant difference at 12 months in clinical response (CGI-C) between patients that had been operated with and without MER (Additional file 1: Table S1).

Focal dystonia patients had significantly longer mean duration of generator life at first replacement than general dystonia patients (*p* < 0.05, Mann-Whitney U test) (Table 2). The number of patients was too small to statistically compare differences for second and third generator replacements. Only one rechargeable IPG was changed during study period, since it depleted completely because of charging difficulties.

**Table 2:** Generator replacement operations

	First	Second	Third
	(*n* = 22)	(*n* = 5)	(*n* = 2)
Generator life ± SD, months			
Focal dystonia	23 ± 5,7 (*n* = 13)	14 ± 0,2 (*n* = 2)	6 (*n* = 1)
Generalized dystonia	15 ± 6,3 (*n* = 5)	19 ± 8,0 (*n* = 3)	26 (*n* = 1)
All	21 ± 7,2 (*n* = 22)	17 ± 6,5 (*n* = 5)	21 ± 7,2 (*n* = 2)
Type			
Medtronic Activa PC	10	3	1
Medtronic Activa RC	12	2	1

In addition one patient had reoperation replacing temporary IPG with Permanent one

There was a significant inverse correlation between DBS voltage setting and generator life, but not with pulse width or frequency at 12 months (r = − 0,566, *p* < 0.05, Spearman correlation) (Additional file 4: Figure S2). The mean stimulation parameters are summarized in Table 3. For all patients, constant-voltage stimulation was used.

**Table 3:** Stimulation parameters

	Focal	Generalized	All
	(*n* = 17)	(*n* = 9)	(*n* = 31)
Amplitude ± SD (V)	2,9 ± 0,8	3,5 ± 0,7	3,0 ± 1,1
Pulse width ± SD (μs)	280 ± 117	246 ± 125	270 ± 115
Frequency ± SD (Hz)	129 ± 22	123 ± 42	130 ± 30
Stimulation type			
Monopolar	12	7	21
Bipolar	3	1	5
Interleave		1	1
Mixed	2		4

There was only one postoperative subclinical intracerebral haemorrhage detected in routine computer tomography control right after the operation. Four patients developed infection leading to the removal of the device. Eleven patients (30%) had postoperative antibiotics, in most cases, the medication was given for mild superficial infections. One patient’s DBS treatment was discontinued after 2 years because of device-related infection and patient’s suboptimal commitment to DBS treatment.

Operation-, hardware- and stimulation-related adverse events are summarized in Table 4. The rate of serious adverse events (SAE) and adverse events (AE) did not differ between age groups, dystonia types or patients that had been operated with and without MER (Additional file 2: Table S2).

**Table 4:** Adverse events in DBS in dystonic patients

	AE^a^	SAE^b^	Resolved^c^
Operation and device-related	n (%)	n (%)	n
Antibiotic use due to wound problem	11 (30%)		11
Infection leading to revision		4 (11%)	4
ICH, subclinical	1 (3%)		1
Device-related revision			
Intracranial electrode revision		2 (5%)	2
IPG malfunction		2 (5%)	2
IPG dislocation		1 (3%)	1
Contact malfunction		1 (3%)	1
Stimulation-related			
Dysarthria	14 (37%)	1 (3%)	7
Dysphagia	4 (11%)	1 (3%)	3
^d^Impaired upper limb coordination	9 (24%)		6
^e^Impaired lower limb coordination	7 (19%)		3
Impaired balance	6 (16%)		2
Neck pain	4 (11%)		4
Anxiety / Depression	3 (8%)		2
Fatique	3 (8%)		1

Symptoms complained by single patients: migraine, double vision and vision blurring, dizziness, and neck pressure, weight loss, hallucinations, tinnitus, and restlessness. *n* = 37. ^a^Adverse event. ^b^Serious adverse event. ^c^Event resolved completely in study period. ^d^inc. hand tremor, micrographia and stifness. ^e^inc. gait stiffness

Altogether four revisions and seven removal operations were done in eight patients.

The most common stimulation related adverse events were dysarthria, impaired upper and lower limb coordination and impaired balance. DBS programming relieved often adverse events, even though the adverse events were not completely resolved in all cases. Adverse events requiring hospital admission occurred in two patients. One patient received a percutaneous gastric tube because of dysphagia, which, however, may also be explained by the progression of the underlying disease. One patient developed severe dysarthria because of DBS that did not respond to routine stimulation parameters and needed a speech communicator. However, the DBS treatment for dystonia was considered beneficial.

## Discussion

DBS is established as an effective treatment for focal, segmental and generalized dystonia [5–7]. Our study assessed the Finnish dystonia DBS patients during 2007–2016. The number of dystonia as well as overall DBS operations has been steadily increasing during the study period. However, in proportion to the total number of DBS operations, dystonia related DBS operations have increased slowly in Finland. No data from other countries for comparison was available.

DBS alleviated dystonia symptoms in most patients, and specifically in patients with isolated cervical dystonia up to 12 months after operation. As compared to the meta-analysis by Moro et al., where the Burke–Fahn–Marsden Dystonia Rating Scale motor score improved 65% in isolated dystonia, our patients had slightly less improvement (55%) [13]. However, the results are not directly comparable and in the meta-analysis better outcome was associated with greater dystonia severity. The majority of our patients had cervical dystonia, with not as high GDS scores and this might limit relative improvement. Only 4 patients did not benefit from DBS. One had progressive underlying disease (NBIA) and two had major side effects with increased stimulation. None of the patients in our study became completely symptom-free. Three patients who returned to work had focal dystonia.

The response to DBS remained at 12 months in focal dystonia patients. According to previous literature, the response has been reported to evolve in the first months after surgery, but the long-term effect seems to be sustained at least 5–10 years [9, 14, 15].

The patients in this study were older in comparison to the meta-analysis by Moro et al., mean age being 50 years compared with 35 years, respectively [13]. As cervical dystonia patients are generally older than generalized dystonia patients, the higher proportion of cervical dystonia probably raises the mean age in this study. The rate of adverse events did not differ between age groups in our study. It has been suggested that earlier DBS interventions result in better outcomes [16]. However, controversial results have been published [17–19]. Also, younger patients may benefit more from DBS [13, 19]. In our study, the duration of disease or age did not correlate with the clinical response in isolated dystonia.

Interestingly, the only patient with post-stroke hemidystonia had a good response. DBS after stroke remains a controversial issue, and there is insufficient data to give general recommendations about it [20].

In this study, about half of patients were referred for DBS once they were already retired or on long sick leave because of dystonia. It remains to be seen, whether earlier intervention in suitable dystonic patients may sustain working ability and reduce social life disabilities.

Present results showed no fatal adverse events or permanent damage. Only one subclinical ICH and four infections leading to revision occurred in our cohort. However, operation and device-related adverse events that led to hospitalization and/or reoperation occurred in eight patients. The type of adverse events was similar to previous studies, but the rate of adverse events was quite high. The occurrence did not differ between dystonia groups or different centres. Previously, the reported rate of adverse events has varied considerably, from 5 to 26%, and in dystonia it is higher than in PD, but comparable to rarer indications like epilepsy and Tourette syndrome [21]. In our patient material, the pulse width was higher than in previous studies, thus this might increase the number of side effects, specifically dysarthria. However, parameter adjustment resolved or relieved adverse events in most cases. In our cohort, technical problems including one electrode revision, were quite common (16%), whereas previously reported lead fractures did not occur [21–23].

The stimulation-related dysarthria and limb coordination problems indicated most probably involvement of internal capsule stimulation. Adverse events were comparable to previous studies [24]. The observed proportion of stimulation related speech impairment was quite high. The result of a recent study suggests that posteriorly located active contacts in GPi may give rise to speech slowing in dystonic patients [25]. Unfortunately, the exact electrode location could not be defined in this study. It remains to be seen, whether the use of directional electrodes can decrease speech problems in patients with dystonia.

Psychiatric or cognitive adverse events were rare, only one patient with previously diagnosed depression complained of the worsening of depression and two other patients reported new onset of depression symptoms postoperatively.

Many centres consider MER as a gold standard for optimal targeting of the DBS lead during operation [26]. However, based on clinical outcome and adverse events, our results do not suggest superiority of MER over macrostimulation in dystonia.

The battery life was somewhat lower than in some previous studies, however, the IPG type was also different [17, 27, 28]. Pulse width was on average higher (270 μs vs. 100–150 μs) while frequency and amplitude were similar in comparison with previous reports [29, 30]. Logically voltage amplitude correlated inversely with battery life, but pulse width did not. Interestingly, the patients with focal dystonia had significantly longer battery life than generalized dystonia patients. The lower voltage values of focal dystonia patients might cause this, even though stimulation parameters did not significantly differ.

Rechargeable IPGs have shown to save costs and reduce infection risk because of fewer replacements compared with non-rechargeable IPGs [27]. Although 27 operations were done due to battery depletion, only 14 rechargeable IPGs were implanted. Most patients were satisfied with the rechargeable battery. However, the dependence on daily charging was the reason for some patients to remain with the non-rechargeable battery.

Being a retrospective uncontrolled observational study, this study comes with some limitations. The placebo effect could not be measured. The exact location of electrodes could not be retrieved from patient databases. Also, the assessment of GDS as well as complication data was based on patient records and not on systematical recording. Furthermore, the follow-up was available for most patients up to 12 months only. The amelioration of motor symptoms was evaluated, but the effects of reduced non-motor symptoms, such as pain, on GDS cannot be completely excluded. Moreover, the total amount of patients was small and especially the groups of generalized and segmental dystonia were heterogeneous, affecting the statistical analysis.

## Conclusion

This study reinforces DBS as an effective treatment option for dystonia, although symptoms are usually not completely eliminated. The treatment is generally safe, and well tolerated after DBS adjustments, but the risk of adverse events needs to be taken into consideration. Present results also suggest that MER is not superior to macrostimulation in dystonia. The number of DBS operations in dystonia patients has grown slower than in other DBS indications in Finland. Thus, more emphasis should be put on increasing awareness of this treatment option in severe drug resistant dystonia.

## Additional files

Additional file 1: **Table S1.** The clinical response as measured with GDS in different patient groups in isolated dystonia. The clinical response as measured with GDS at 6 and 12 months with isolated dystonia patients did not differ between age groups under vs. over 50 years, duration of disease under vs. over 10 years, patients with vs. without previously diagnosed depression or that had been operated with and without MER. (DOCX 13 kb)

Additional file 2: **Table S2.** The number of patients in different groups with AE and SAE. The rate of AE and SAE did not differ between age groups, dystonia types or patients that had been operated with and without MER. (DOCX 13 kb)

Additional file 3: **Figure S1.** The annual number of DBS operations in different dystonia types and total DBS operations in Finland. The number of DBS operations in dystonia are shown as stacked column graphs and total number of all DBS operations marked as line. The scale for number of dystonia DBS is on left and for total DBS operations on right. (TIF 811 kb)

Additional file 4: **Figure S2.** Scatter plot of generator life. Scatter plot of generator life versus amplitude (A), pulse width (B) and frequency (C), All cases were plotted individually. Black line represents regression line. There was a significant inverse correlation between DBS voltage setting and generator life, but not with pulse width or frequency at twelve months (r = − 0,566, *p* < 0.05, Spearman correlation). (TIF 1504 kb)

## Data Availability

The datasets analyzed during the current study are available from the corresponding author on reasonable request.

## Abbreviations

BoNT: Botulinum neurotoxin
DBS: Deep brain stimulation
GDS: Global Dystonia Severity Rating Scale
GPi: Globus pallidus interna
MER: Microelectrode recording
NBIA: Neurodegeneration with brain iron accumulation
PD: Parkinson’s disease
