# Supplementary material for: Deep brain stimulation for dystonia in Finland during 2007–2016
Source: BMC Neurol. 2019 Jun 24;19:137. doi: 10.1186/s12883-019-1370-y (PMC6589889; doi:10.1186/s12883-019-1370-y)
Supplement: Supplementary file 1 — Table S1. The clinical response as measured with GDS in different patient groups in isolated dystonia. The clinical response as measured with GDS at 6 and 12 months with isolated dystonia patients did not differ between age groups under vs. over 50 years, duration of disease under vs. over 10 years, patients with vs. without previously diagnosed depression or that had been operated with and without MER. (DOCX 13 kb) [file 12883_2019_1370_MOESM1_ESM.docx]

Online only

| Supplement table 1. the clinical response as measured with GDS in different | | |  |
| --- | --- | --- | --- |
| patient groups in isolated dystonia. | |  |  |
|  | preoperative | 6 months | 12 months |
| Age under 50 years | 8,0 ± 2,5 (n=14) | 5,2 ± 3,2 (n=13) | 3,2 ± 2,0 (n=11) |
| Age over 50 years | 8,1 ± 3,2 (n=16) | 4,8 ± 3,6 (n=14) | 3,2 ± 2,2 (n=14) |
|  |  |  |  |
| Disease duration under 10 years | 8,3 ± 2,0 (n=13) | 6,1 ± 3,4 (n=13) | 3,4 ± 1,9 (n=11) |
| Disease duration over 10 years | 7,9 ± 3,4 (n=17) | 3,9 ± 3,1 (n=14) | 3,1 ± 2,3 (n=14) |
|  |  |  |  |
| Depressed | 6,9 ± 2,3 (n=9) | 5,3 ± 3,0 (n=9) | 3,0 ± 2,1 (n=7) |
| Not depressed | 8,6 ± 3,0 (n=21) | 4,8 ± 3,6 (n=18) | 3,2 ± 2,1 (n=18) |
|  |  |  |  |
| MER used | 8,6 ± 3,8 (n=17) | 5,9 ± 3,5 (n=15) | 4,4 ± 3,6 (n=15) |
| MER not used | 9,8 ± 5,3 (n=20) | 5,1 ± 3,3 (n=19) | 3,5 ± 3,1 (n=16) |
