# Supplementary material for: Deep brain stimulation for dystonia in Finland during 2007–2016
Source: BMC Neurol. 2019 Jun 24;19:137. doi: 10.1186/s12883-019-1370-y (PMC6589889; doi:10.1186/s12883-019-1370-y)
Supplement: Supplementary file 2 — Table S2. The number of patients in different groups with AE and SAE. The rate of AE and SAE did not differ between age groups, dystonia types or patients that had been operated with and without MER. (DOCX 13 kb) [file 12883_2019_1370_MOESM2_ESM.docx]

Online only

| Supplement table 2. The number of patients with adverse event and serious adverse event | | | | |
| --- | --- | --- | --- | --- |
|  | AE* | | SAE† | |
|  | n ( %) | | n ( %) | |
|  | Infection | Other | Infection | Other |
| Dystonia type |  |  |  |  |
| Focal | 6 (29%) | 9 (43%) | 1 (5%) | 5 (24%) |
| Generalized | 3 (30%) | 6 (60%) | 1 (10%) | 1 (10%) |
| Segmental | 1 (20%) | 3 (60%) | 1 (20%) |  |
| Hemidystonia | 1 (100%) |  | 1 (100%) | 1 (100%) |
|  |  |  |  |  |
| Age |  |  |  |  |
| 20-40 years | 3 (75%) | 1 (25%) | 2 (50%) | 2 (50%) |
| 40-60 years | 6 (25%) | 13 (54%) | 1 (4%) | 5 (21%) |
| Over 60 years | 2 (25%) | 4 (50%) | 1 (12,5%) |  |
|  |  |  |  |  |
| MER used | 7 (41%) | 10 (59%) | 2 (12%) | 3 (18%) |
| MER not used | 4 (20%) | 11 (55%) | 4 (20%) | 1 (5%) |
| *adverse event. † serious adverse event. | | |  |  |
