# Supplementary figures and images for: Deep brain stimulation for dystonia in Finland during 2007–2016
Source: BMC Neurol. 2019 Jun 24;19:137. doi: 10.1186/s12883-019-1370-y (PMC6589889; doi:10.1186/s12883-019-1370-y)

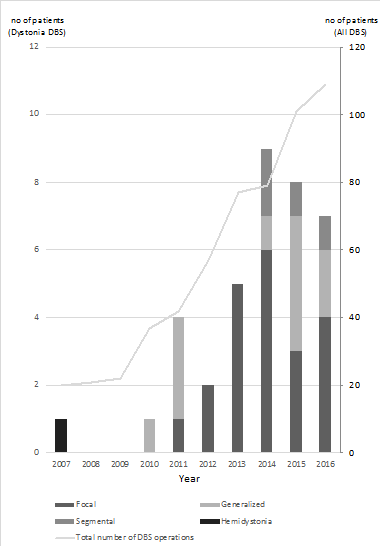

Supplement: Supplementary file 3 — Figure S1. The annual number of DBS operations in different dystonia types and total DBS operations in Finland. The number of DBS operations in dystonia are shown as stacked column graphs and total number of all DBS operations marked as line. The scale for number of dystonia DBS is on left and for total DBS operations on right. (TIF 811 kb) [file 12883_2019_1370_MOESM3_ESM.tif]

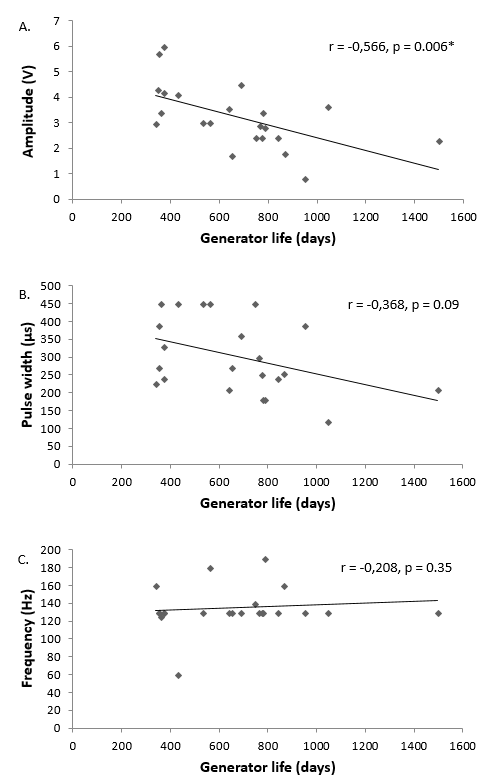

Supplement: Supplementary file 4 — Figure S2. Scatter plot of generator life. Scatter plot of generator life versus amplitude (A), pulse width (B) and frequency (C), All cases were plotted individually. Black line represents regression line. There was a significant inverse correlation between DBS voltage setting and generator life, but not with pulse width or frequency at twelve months (r = − 0,566, p < 0.05, Spearman correlation). (TIF 1504 kb) [file 12883_2019_1370_MOESM4_ESM.tif]
